# Supplementary material for: Development of a computer-aided design software for the quantitative evaluation of aesthetic damage
Source: PLoS One. 2019 Dec 18;14(12):e0226322. doi: 10.1371/journal.pone.0226322 (PMC6919621; doi:10.1371/journal.pone.0226322)
Supplement: S1 Code site — 1. (ZIP) [file pone.0226322.s001.zip › [EESC_jr][Projeto]Relatorio_07_21/reports/templates_zip/gentelella-master/vendors/google-code-prettify/examples/quine.html]

Making Quines Prettier


# Making Quines Prettier

Below is the content of this page prettified. The `<pre>`
element is prettified because it has `class="prettyprint"` and
because the sourced script loads a JavaScript library that styles source
code.

The line numbers to the left appear because the preceding comment
`<?prettify lang=html linenums=true?>` turns on
line-numbering and the
stylesheet
(see `skin=sunburst` in the `<script src>`)
specifies that every fifth line should be numbered.

prettify lang=html linenums=true?
